# The needs for medical visit accompaniment services among older patients with chronic diseases and their family members: a qualitative study

**DOI:** 10.3389/fpubh.2025.1577329

**Published:** 2025-05-09

**Authors:** Yu-Han Chen, Jia-Ying Zhu, Qin-Ying Fu, Xin-Ye Yu, Chang-Hong Wu, De-Qin Huang

**Affiliations:** ^1^School of Public Health and Nursing, Hangzhou Normal University, Hangzhou, China; ^2^Alibaba Business School, Hangzhou Normal University, Hangzhou, China

**Keywords:** outpatient, older patients, chronic diseases, medical visit accompaniment, needs, qualitative study

## Abstract

**Objective:**

To explore the specific needs of older patients with chronic diseases and their family members for medical visit accompaniment services in outpatient settings.

**Methods:**

The interview outline was developed based on the theory of existence, relatedness, and growth needs. Semi-structured in-depth interviews were conducted with older patients and their family members. Directed content analysis was used to analyze the interview data. This study followed the Consolidated Criteria for Reporting Qualitative Research (COREQ) guidelines to report the study.

**Results:**

Nineteen older patients with chronic diseases and 17 family members, were interviewed in the outpatient clinic of a tertiary hospital in Hangzhou from July to August 2024. Four themes emerged with ten sub-themes: (1) existence needs (basic services, security, and the qualifications of medical visit accompaniment providers), (2) relatedness needs (relationships with medical visit accompaniment providers and emotional support), (3) growth needs (willingness to learn and learning contents), and (4) the need for optimizing service provision (improving the efficiency of medical visits, ensuring transparency of the visit, and delivering personalized services).

**Conclusion:**

The needs of older patients with chronic diseases for medical visit accompaniment services included existence needs, relationship needs, growth needs, and the need for optimizing service provision. The government and institutions providing medical visit accompaniment services should conduct qualification assessment, enhance personnel management, and improve the services as to effectively promote the medical experience and healthy ageing of older patients.

## 1 Introduction

Ageing has become one of the most significant global trends of the 21st century. According to the World Population Prospects 2024 (1), the global population aged 65 and older is expected to reach 2.2 billion by the late 2070s, exceeding the number of individuals under the age of 18. As the country with the largest population of older adults (defined as those aged 60 and above) in the world (2), the number of older people in China has reached 296 million by 2023, accounting for 21.1% of the total population (3). The huge older population has brought tremendous pressure on family, the social security system, and the healthcare system.

The older population is more likely to suffer from chronic diseases, the high-incidence of which include cardiovascular diseases, diabetes, chronic obstructive pulmonary diseases, etc. These diseases often have prolonged courses and require multiple medical treatments. However, studies indicated that communications between older patients and healthcare providers were often negatively affected by a number of factors, including the patients’ cognitive impairment or physical dysfunction (4), the lack of family accompaniment during consultations (5), and language barriers (6). Moreover, older patients with comorbidities in Western countries face more difficulties when seeking medical treatment. For example, they often need to coordinate visit to different outpatient departments (7). Long waiting time for medical treatment (8) also increases the difficulty for older patients to medical visit. In China, the first stop for diagnosis and treatment of older patients with chronic diseases is outpatient clinics (9). In recent years, with the improvement of the information technology in Chinese hospitals, multi-functional self-service registration machines, electronic appointment and registration platforms, and convenient mobile payment methods have been employed in outpatient services. However, due to the fact that older patients learn and adapt to new technologies relatively slowly than younger ones, they generally encounter barriers when receiving high-tech outpatient services (10). Barriers to healthcare access for older patients are becoming increasingly prominent worldwide, directly limiting the timely satisfaction of their health needs. As a result, worsened health conditions occur, thus increasing the physical and mental burden, impacting the quality of life, and even threatening the safety of life of older patients (11). Ultimately, the healthy ageing process of the older population will be impeded.

As patient-centered health services, medical visit accompaniment (MVA) services can effectively cope with the difficulty in medical visits for older outpatient patients (12). In the United States, family members such as spouse and children of older patients typically serve as medical visit accompaniment providers (MVAPs) (13). They participate in the entire medical visit process: assisting them in transportation, exchanging information with doctors, and follow-ups. Ishikawa et al. (14) found that the active participation of MVAPs greatly improved the efficiency of medical communication; as well as patients’ and doctors’ satisfaction with visits, appointment and treatment compliance. However, studies on MVA in Western countries mainly focused on the duties of the spouses, children of older patients or volunteers in community (12, 14). On one hand, it is lacking of exploration of the MVA needs of older patients with chronic diseases; on the other hand, only few studies reported occupational MVAPs.

Chinese occupational MVA service is still in its infancy, however, its great potential in solving the problem of difficult consultation among older patients with chronic diseases cannot be ignored. In China, Medical visit accompaniment providers (MVAPs) refer to individuals who offer companionship and assistance to patients during medical visits as their occupation (15). Their responsibilities include helping older patients with registration, waiting for consultation and examination, receiving medical reports, etc. (16). Research showed that Chinese people have an urge for MVA services and prevailingly hold a positive attitude towards MVAPs (17). Most studies on MVA in China focus on the perceptions and willingness of older patients and their family members regarding MVA services. However, research on the specific needs of older patients and family members for MVA is limited, which may affect the quality of MVA services and the medical experience of older patients.

Hence, this study explored the specific needs of outpatient older patients with chronic diseases and their family members for MVA services, aiming to provide insights for promoting the medical experience and healthy ageing of older patients.

## 2 Methods

### 2.1 Design

The study utilized a descriptive qualitative research methodology, and the research group adopted the Consolidated Criteria for Reporting Qualitative Research (COREQ) guidelines (18), to ensure that the study meets the recommended standards for reporting qualitative data (see Supplementary File 1).

### 2.2 Study setting and recruitment

The purposive sampling method was used to select outpatient older patients and their families from a tertiary hospital in Hangzhou from July to August 2024. The inclusion criteria for outpatient older patients were: (1) Age ≥ 60 years old; (2) Diagnosed with at least one chronic disease; and (3) Informed consent for the study. The exclusion criteria were: (1) Severe cognitive dysfunction; (2) Severe physical diseases; (3) Severe communicative problem such as hearing and/or speech dysfunction; and (4) Severe mental disorders (e.g., schizophrenia, bipolar disorder). The inclusion criteria for family members were: (1) Having one or more older family members (≥60 years old) with at least one chronic disease diagnosed; (2) Able to communicate; and (3) Informed consent to the study. The exclusion criteria were: (1) Suffering from serious physical diseases; (2) Serious mental disorders (e.g., schizophrenia, bipolar disorder); and (3) Serious hearing, speech, and cognitive dysfunction. The sample size was based on information saturation. When no new themes emerged, the researchers stopped interviewing (19).

### 2.3 Theoretical framework

Based on Maslow’s hierarchy of needs theory, Alderfer proposed the Existence-Relatedness-Growth theory (the ERG theory), which identified that human have three core needs: needs for existence (E), relatedness (R), and growth (G) (20). Using the ERG theory, the outline of interviews was designed.

(a) Existence needs are the basic condition of material life related to physiology, mainly including the needs for health and security. Reflected in interview questions about basic services (e.g., What services would you like an MVAP to provide for you/your older family member?); safety concerns (e.g., What are your concerns or worries during the MVA process?); and MVAPs qualifications (e.g., What requirements do you have for MVAPs?).
(b) Relatedness needs are the desire to maintain friendly relations with other people. Reflected in interview questions exploring emotional support and expected relationships with MVAPs (e.g., What kind of relationship would you prefer with a MVAPs?).
(c) Growth needs are people’s inner desire to be developed. Reflected in interview questions about the willingness to learn (e.g., What would you like to learn during MVA services? or What would you like a MVAP to teach your older family member while having MVA service?).

### 2.4 Data collection

Before conducting formal interviews, the research team carried out pre-interviews with 4 outpatient older patients with chronic diseases and 4 family members of older patients. Based on the results of the pre-interviews and advice of experts, the interview outline was revised and optimized. Semi-structured in-depth interviews were then conducted to collect data by face-to-face. Before interviewing, researchers established a relationship with the interviewees. Researchers informed all interviewees of the purpose, significance, and methods of the study, promised confidentiality, and obtained their consent on recording the interviews. All interviewees signed informed consent forms and voluntarily participated in the study. The interviews were conducted in a conference room in the outpatient clinic to ensure quietness and privacy. During the interviews, the researchers avoided inducing conversation and paid attention to and recorded the nonverbal behaviours of the participants, such as facial expressions, tone of voice, and gestures. At the end of the interviews, the participants were given a brief reminder, and the interviews were concluded. The main interview questions (Table 1) were as follows.

**Table 1:** Interview outline based on ERG theory.

Dimension of ERG theory	Question
Introductory phase	(1) Please talk about your (your older family member’s) experience on the most recent medical visit. (2) What difficulties have you (your older family member) encountered during the medical visit?
Existence needs/relatedness needs	(3) What services would you like an MVAP to provide for you (your older family member)? (4) What are your concerns or worries during the MVA process? (5) What requirements do you have for MVAPs? (6) What kind of relationship would you prefer with an MVAP?
Growth needs	(7) What would you like to learn during the MVA service? / What would you like a MVAP to teach your older family member during the MVA service?
Closing phase	(8) What suggestions or advice do you have about this interview?

### 2.5 Data analysis

After each interview, the researchers transcribed the audio recordings and field notes into text and compared them with the original recordings to ensure accuracy and completeness. Older patients were coded as N1–N19 and family members as S1–S17 and their separate Word documents were created. The MAXQDA 2022 was used to store and manage the qualitative data obtained. Interview data were analyzed using directed content analysis (21). The specific steps were as follows: (1) Key concepts were selected as initial coding categories based on ERG theory and literature. (2) By repeatedly reading the transcripts, the two researchers focused on the content related to the research question or ERG theory, labeling and annotating the contents, and extracting semantic units. (3) Codes were created by categorizing the relevant semantic units using the predetermined coding categories. (4) After completing coding, the researcher determined whether subcategories needed to be constructed based on the similarities and differences between the codes. (5) Texts that could not be classified using the initial coding scheme were given new codes, which ultimately led to the formation of new category genera, expanding the ERG theory.

The research team consisted of six researchers who had experiences in qualitative study. CHY, ZJY, FQY, and WCH conducted the interviews and took notes. CYH and ZJY independently analyzed the data of older patients while FQY and WCH independently analyzed the data from family members of older patients, with discrepancies resolved through consensus with HDQ. CHY, ZJY, FQY, YXY are female nursing undergraduates, WCH is a male undergraduate majoring in electronic commerce. HDQ, the lecturer from a nursing school, is a ph. D in nursing. HDQ trained other researchers in this study about how to conduct the qualitative study. The researchers avoided assumptions before collecting and analyzing the interviewing data.

### 2.6 Ethical considerations

This study was approved by the Ethics Committee of Hangzhou normal University, No. 2023089, and the Ethics Committee of The Affiliated Hospital of Hangzhou normal University, No. 2023(ES)-KS-162, respectively.

### 2.7 Rigor and reflexivity

The trustworthiness of this study was based on the four criteria for qualitative research proposed by Lincoln and Guba: credibility, transferability, dependability, and confirmability (22). To enhance the credibility of this study, we adopted triangulation in data sources, peer debriefing and member checking. Triangulation was achieved through collecting interview data from both older adults and family members. Peer debriefing was conducted with a professor, a qualitative research expert outside the research team, who provided an external check to ensure the rigor of the results. As for member checking, the interviewees were asked to clarify any unclear information in real time during the interviews. After the interviews, transcripts were later returned to the interviewees for further confirmation. Revisions were made based on their feedback to ensure the authenticity and accuracy of the data. Detailed descriptions were used to ensure transferability, including vivid narratives, specific examples, and direct quotations from data sources such as interviews and memoranda. To ensure dependability, the research process was examined by two experts outside the research team, to assess potential researcher bias and the appropriateness of the research design. The data, findings, interpretations, and inferences were thoroughly reviewed by two experts, both external to the research team to achieve confirmability.

## 3 Results

### 3.1 Characteristics of participants

**Figure 1:**
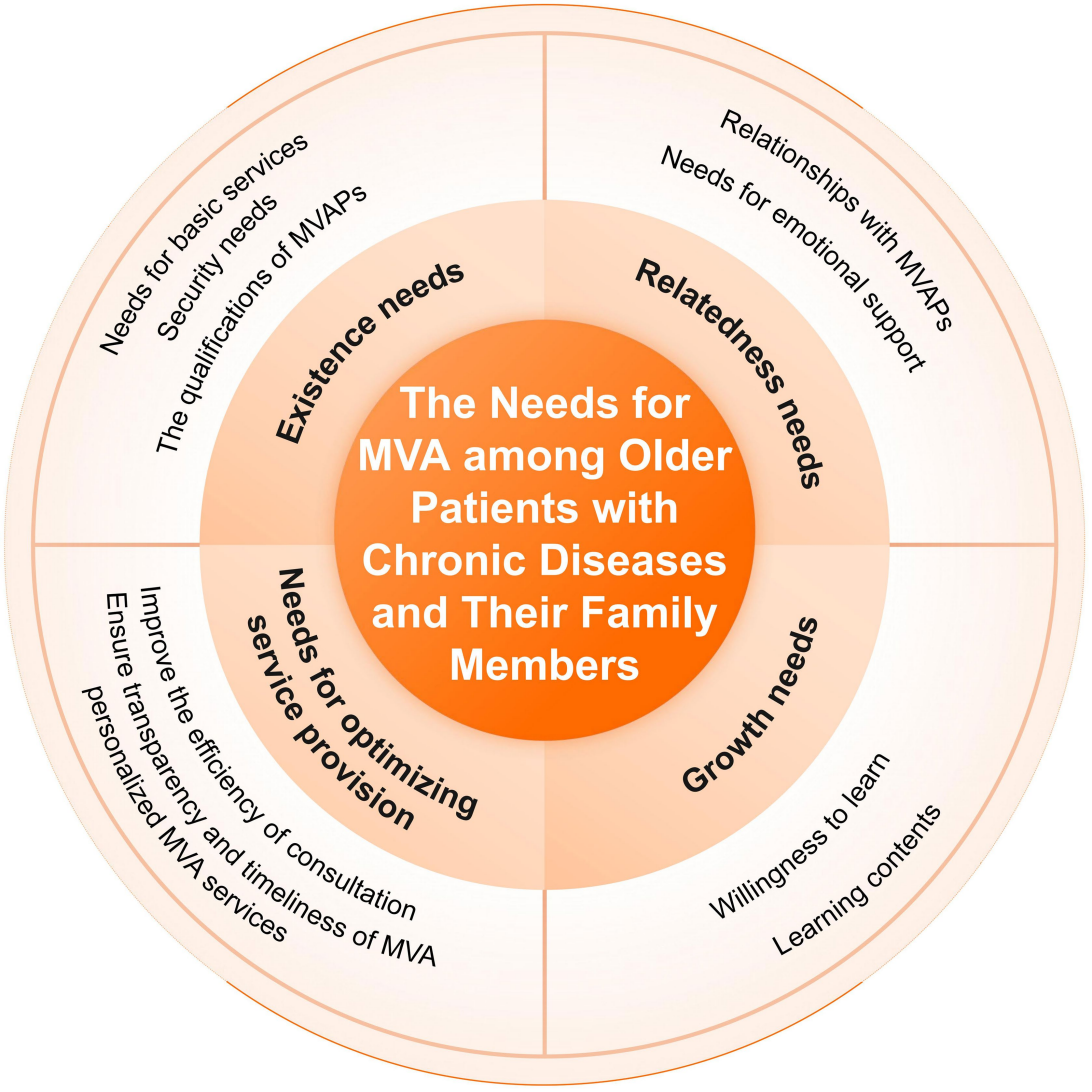
An overview of the four themes describing the needs of outpatient older patients with chronic diseases and their family members for MVA services.

The final sample included 19 outpatient older patients with chronic diseases, comprising 8 males and 11 females aged between 60 and 85 years (mean age:72.5 ± 12.5 years). Additionally, 17 family members of older patients participated, including 6 males and 11 females, aged between 18 and 55 years (mean age: 36.5 ± 18.5 years). Detailed demographic information were shown in Tables 2, 3. An overview of the four themes describing the needs of outpatient older patients with chronic diseases and their family members for MVA services is presented in Figure 1.

**Table 2:** Basic information of outpatient older patients with chronic diseases.

Characteristic	Percentage (%)	Number of participants
Gender		
Male	42.11	8
Female	57.89	11
Age (years old)		
60–74	63.16	12
75–85	36.42	7
Educational background		
Illiteracy	5.26	1
Primary and below	26.32	5
Junior high school	31.58	6
High school or technical secondary school	21.05	4
College	15.79	3
Number of chronic diseases		
1	68.42	13
2	26.32	5
3	0	0
4	5.26	1
Monthly income (yuan)		
<1,000	5.26	1
1,000–1999	5.26	1
2000–3,999	31.58	6
4,000–5,999	31.58	6
≥6,000	26.31	5
Living arrangement		
With children	26.31	5
With spouse	63.16	12
With spouse and children	5.26	1
Alone	5.26	1
Frequency of medical visit		
Once a week	5.26	1
Once a month	73.68	14
Once a quarter	5.26	1
Once or twice a year	10.52	2
Every few years	5.26	1
Source of income		
Pension	80	16
Child support	20	4
Medical expenditure		
Self-financing	20	4
New rural cooperative medical system (NCMS)	15	3
Employee insurance	35	7
Medical insurance for urban residents	30	6
Marital status		
Divorce	5.26	1
Married	89.47	17
Widowhood	5.26	1
Number of children		
One child	52.63	10
Two children	31.58	6
Three children	10.52	2
Four or more children	5.26	1
Occupational status		
Unemployed	15.79	3
Retirement	84.21	16
Whether or not having received MVA services		
Yes	15.79	3
No	84.21	16

**Table 3:** Basic information of family members of outpatient older patients with chronic diseases.

Characteristic	Percentage (%)	Number of participants
Gender		
Male	35.29	6
Female	64.71	11
Age (years old)		
18–44	82.35	14
45–59	17.65	3
Educational background		
Junior high school	11.76	2
High school or technical secondary school	5.88	1
Collegiate	52.94	9
Graduate students or above	29.41	5
Marital status		
Married	64.71	11
Unmarried	35.29	6
Having siblings		
Yes	64.71	11
No	35.29	6
Monthly income (yuan)		
<2,999	17.65	3
3,000–4,999	5.88	1
5,000–6,999	5.88	1
7,000–9,999	29.41	5
≥10,000	41.77	7
Frequency of medical visit for older patients		
Once a month	23.53	4
Once a quarter	23.53	4
Semi-annually	29.41	5
Once a year	23.53	4
Medical expenditure for older patients		
Self-financing	15.79	3
Medical insurance for urban residents	42.11	8
New rural cooperative medical system (NCMS)	15.79	3
Employee insurance	26.31	5
Relationship with older patient		
Grandchildren	35.29	6
Son or daughter	64.71	11
Occupational status		
Student	11.76	2
Employed	88.24	15
Whether or not having received MVA services		
Yes	17.65	3
No	82.35	14

### 3.2 Existence needs

#### 3.2.1 Needs for basic services

The needs for basic services encompassed the duties before, during, and after the medical consultation process. Specifically, the duties before accompanying patients involved understanding the patients’ conditions and preparing necessary medical aids.

“I think they should first know my condition, what kind of illness I’m seeking consultation for today, and then, such as wheelchairs, these must be fully equipped, even if it is a wheelchair rented outside the hospital, I think it should be provided” (N2).

“(MVAPs) need to communicate with the family members first and discuss precautions before accompanying the older patients. For example, if the family informs them that the older patient has poor hearing, they can choose to communicate using written text …” (S16).

The service needs during the medical consultation included assisting in registration, interpreting the doctor’s instructions, acting as a guide, and assisting in examination and treatment.

“(MVAPs) can help with registration, because I don’t know how to register or how to get a row number, I’ve never done it myself before” (N8).

“If the older patient is educated, MVAPs can write the information on the package of the medicine, for example, if it should be taken three times a day, you can write a ‘3’ or ‘2’ on the packaging …” (N3).

“Apart from registering, older patients may not know where to go for blood samples or other examinations. As for me, I don’t know how to go through the whole process, and I don’t know where to go. It is necessary for the MVAPs to guide older people to the right location” (S13).

Family members expressed a need for services after the medical consultation: they expected MVAPs to provide accurate feedback on the results of the consultation and to provide reminders for next visit. Older patients had not yet mentioned these needs.

“How can you explain the doctor’s order in a right way? Older patient may be unable to make plans or follow the doctor’s order on their own, which require the cooperation of their children. In this case, giving feedback to older patients’ children, MVAPs serve as a valuable intermediary” (S4).

“Then the best thing is that MVAPs call regularly to remind older patients and their family members the scheduled time of next visit, the approximate duration of drug treatment, medication and nursing requirements and other such details. Another important role would be to keep timely record …” (S10).

#### 3.2.2 Security needs

Security needs played a crucial role in the treatment of older patients with chronic diseases, primarily involving the ability to respond effectively to emergencies during MVA services provision. Older patients expected MVAPs to remain calm in unexpected situations such as falls and heart attack, use first-aid skills and contact their family members quickly. Family members hoped that MVAPs could contact medical teams to deal with the accident, so that the older patients could receive timely and effective treatment.

“(If there is an accident,) they should quickly tell patient to sit down and have a rest. Some first aids should be done quickly, conducting CPR. Conducting first aid while notifying the family member and giving him some water or other assistance as needed at the same time” (N2).

“If the older patient falls, the MVAP should immediately seek help from a doctor to provide first aid” (S5).

#### 3.2.3 The qualifications of MVAPs

The qualifications of MVAPs, as identified by older patients and their family members, included professional knowledge, official certification, and both physical and mental qualities proposed by older patients and family members.

“A MVAP should have some basic medical knowledge. If I catch a cold, they should know I might have a fever or seizure … they should know which department I should go to, if the older patient have a heart attack, they should (guide me) to go to the cardiovascular department … for gastric pain, they (should guide me) go to the gastroenterology department. These are things they should generally be familiar with” (N10).

“I would consider whether the MVAP holds the relevant certificates, and whether their platform is authorized and licensed by a recognized certification body” (S7).

“Another essential quality for MVAPs is physical strength. They need to be able to assist older patients in tasks such as climbing stairs; otherwise, it could become quite difficult” (N1).

“Another thing is that the MVAPs need to have a good mentality, and if they are vulnerable, they are not competent … They need to be mentally prepared, for example, if something happens half-way, they should also have a good ability to deal with the accidents, such as dealing with the accidents calmly in a right way” (S1).

### 3.3 Relatedness needs

#### 3.3.1 Relationships with MVAPs

Many outpatient older patients with chronic disease expressed that they desired to get along with their MVAPs as family members or friends.

“Getting along like family members is mostly desirable, and like friends is great as well” (N1).

“Well, getting along like family members. Um, okay. Um, well, like family members … in this way taking care of us quite well” (N17).

Some family members preferred that MVAPs to maintain the relationship with older patients like friends or regard them as clients.

“Being a friend is fine, there is no difference, just be friends. Meanwhile, different people hold different ideas about the relationships, and it depends on the older patients’ needs” (S18).

“I think it depends, because different people have different needs, some people feel that he is quite happy when you talk to him, some people feel that you are annoying when you talk too much” (S12).

#### 3.3.2 Needs for emotional support

A number of older patients interviewed strongly proposed that MVAPs should provide companionship and chat services.

“Waiting is boring and we can talk to each other about the disease or other topics” (N16).

“Haha, just chatting is fun. It feels good to have a conversation” (N17).

Most family members believed that MVAPs should communicate with the older patients or provide them with psychological support to alleviate their anxiety while they were waiting for treatment.

“MVAPs should professionally explain the patient’s condition, reassuring him that it may not lead to serious consequences, and help to relieve his stress appropriately” (S2).

“First understand the older patient’ conditions and then give him/her psychological comfort accordingly” (S9).

### 3.4 Growth needs

#### 3.4.1 Willingness to learn

Many older patients with chronic diseases expressed a desire to fulfill their self-development needs by learning more about their health conditions and becoming familiar with the consultation process.

“Learning how to take medicine” (N1).

“I wish I could learn how to register, getting the medication, and so on” (N2).

However, a notable proportion of family members believed that older patients with chronic diseases did not require additional learning.

“Not really need… I don’t need to teach him… Well, it’s not necessary …” (S10).

“For older people, that’s not necessary, they shouldn’t have to learn anything” (S11).

#### 3.4.2 Learning contents

This study found that the learning contents mainly included acquiring knowledge about diseases and understanding how to navigate the medical consultation process.

Older patients were often able to propose detailed learning needs based on their own conditions, such as learning about disease prevention.

“I come here to check my eyes today. I want to know some information related to my eyes, and how I can prevent the eye disease in the future …” (N1).

“Instruction, prevention, what we should do in the future. Please instruct me if you can” (N9).

In contrast, family members tended to give suggestions from the perspective of bystanders, and their expression of needs is relatively vague, lacking a deep understanding of the older patients’ learning needs.

“Some other knowledge related to this disease, you can teach us” (S13).

“I think it’s better to remind older people of simple things and explain knowledge to them in an easy way” (S14).

Regarding the consultation process, a subset of older patients and family members expressed interest in learning how to register, how to pay, and how to find their way in the hospital.

“Let him (the older patient) familiarize himself with the procedures for seeking medical treatment in the hospital … learn how to register and pay” (S7).

“Of course, I want to learn. I want to learn how to recognize the hospital’s paths and how to get to different departments” (N2).

### 3.5 Needs for optimizing service provision

The results of the interviews showed that, in addition to the three major needs of existence, relatedness, and growth, the family members, in particular, put forward corresponding needs: needs for improving the efficiency of consultation, ensuring transparency and timeliness of MVA, and personalized MVA services.

Some family members urgently hoped that the MVAPs would improve the efficiency of the consultation by adjusting schedules to rationally shorten the waiting time for medical visits; while the interviewed older patients did not report the above needs.

“And then if MVA services could help shorten the visit time, that would be very practical” (S2).

“MVAPs would be able to use the time more rationally, in this way we won’t waste time and energy …” (S4).

Most interviewed family members preferred that the MVAPs could ensure the transparency and timeliness in service delivery, so that they could stay informed about the medical visits of their older relatives.

“MVAPs need to report to the family members, for example, on the physical conditions of this older patient today” (S3).

“So, I hope that when you accompany the patient, you record the visit. Otherwise, there may be discrepancies when you communicate with family members … If it’s recorded, the patient’s children can listen to it afterward” (S8).

Both older patients and family members expressed a need for receiving personalized MVA services.

“For example, I am consulting my eye problems today. I want to know the cause of my eye discomfort and what I need to pay attention to” (N2).

“The service should be more in-depth. Ideally, we prefer to recommending that MVAPs are specialists, for example, patients with cardiovascular problems have companions specialized in cardiovascular …” (S4).

## 4 Discussion

This study indicated that the needs of outpatient older patients with chronic diseases and their family members for MVA services are consistent with the ERG theoretical framework, which states that people’s needs include existence, relatedness, and growth needs. In addition, the study found that older patients and their family members also had special needs for MVA services, including improving the efficiency of medical visits, enhancing the transparency of MVA service delivery, and providing personalized services. This is consistent with the statement proposed by the ERG theory that “an individual can have more than one need simultaneously” (20).

### 4.1 Existence needs: fundamental requirements for MVA services

The results showed that the existence needs of outpatient older patients with chronic diseases and their family members included needs for basic services, security needs and the qualification of the MVAPs.

#### 4.1.1 Continuum of basic services needs in MVA services

The needs for basic services, which were the main content of the MVA, ran through the entire MVA service process—before, during and after medical visits. These needs involved understanding the patient’s conditions in advance, assisting in registration, assisting in medication and examination and explaining the doctor’s instructions, etc. This finding is in line with previous studies (12, 23). Although those studies primarily focused on older patients’ family members, friends, or volunteers, their responsibilities closely resemble the existence needs identified in this study. This suggests that occupational MVAPs can enhance the quality of their services by drawing on the practices and roles traditionally undertaken by informal caregivers to offer more thoughtful and personalized care.

#### 4.1.2 Security needs: first aid training and emergency response

The security needs for MVA services were mainly in coping with accidents. Chen et al. (24) emphasized the importance of safeguarding patients’ lives in the process of MVA, pointing out that the administrators of MVA services need to strengthen the training of MVAPs about first aid skills, including cardiopulmonary resuscitation. Consequently, the legitimate rights and lives of older patients could be protected. In South Korea, the Medical Dispute Mediation and Arbitration System establishes an emergency response plan, and immediately starts an early warning or alarm mechanism once an abnormal situation is found, effectively guaranteeing the safety of older patients (25). Currently, the Ministry of Human Resources and Social Security in China has incorporated first aid training into the standardized curriculum for MVAPs, covering essential skills such as CPR, handling falls, and managing acute symptoms like chest pain or dizziness (26). These equip MVAPs with the necessary competencies to address potential emergencies during medical visits, thereby enhancing patient safety.

#### 4.1.3 Professional qualification requirements for MVAPs

MVAPs were expected to demonstrate professional competence, including possessing medical or nursing knowledge, relevant certificates, effective communication skills, and both physical and mental qualifications. Previous studies have found that older patients are often accompanied temporarily by their spouses, children, other relatives, friends and even community volunteers, which means that they have no clear qualification requirements for accompanying patients. Thus, they just fill the vacancy of accompanying patients (13, 23). However, this study found that older patients with chronic diseases and their families were more inclined to be accompanied by MVAPs who had undergone professional training, held certifications and were familiar with medical knowledge. While MVA services are predominantly implemented across Asian countries, most of these services have not yet been deployed by unified institutions or departments (27). For example, MVAPs in Japan are typically patient’s family members and lack professional qualifications (28). In China, although institutions offering MVA services are gradually emerging, the industry remains in an early stage. A significant proportion of MVAPs still lack professional qualifications, contributing to limited public acceptance of their services (29).

Another key finding of this study was that the interviewees generally emphasized the importance of MVAPs possessing both physical and psychological qualities. This may be due to the fact that, on one hand, MVAPs are required to perform physical tasks, such as carrying older patients up and down stairs and carrying objects; on the other hand, they need to show sufficient patience, empathy, and emotional management skills as older patients experience physical decline, decreased visual abilities, cognitive decline and slow reactions (30). In recent years, under the organization of the Ministry of Human Resources and Social Security, the Chinese government has gradually trained a group of qualified MVAPs to meet the needs of patients and their family members (26). However, there is still a large gap in the number of professional MVAPs at present. Moving forward, it is crucial for government departments to provide policy guidance and continuously expand the scale of professional training to ensure a solid talent base for the industry’s development.

### 4.2 Relatedness needs: social and emotional dimensions

#### 4.2.1 Relationship dynamics with MVAPs

The relationship needs of older patients with chronic diseases and their families in outpatient clinics included the relationship with the MVAPs and needs for emotional support. Regarding their relationship with accompaniment providers, older patients expressed a desire to connect with MVAPs like a family member or friend, while family members preferred that MVAPs maintain a more formal relationship, akin to that of an employee or friend. This finding indicated that older patients sought closer emotional bonds with MVAPs, which aligns with the Socioemotional Selectivity Theory (31). The theory proposed that emotional goals become increasingly significant with age, leading older adults to prioritize emotionally meaningful social partners compared to younger individuals. In contrast, family members exhibited an instrumental relationship orientation consistent with the cost–benefit analysis framework of Social Exchange Theory (32). Accordingly, MVAPs should adopt differentiated strategies: providing emotion-focused support for older patients while maintaining professional boundaries and delivering standardized services for family members.

#### 4.2.2 Emotional support strategies for MVAPs

Studies have found that older adults with chronic diseases were prone to loneliness and social isolation (23). Coupled with the unfamiliar hospital environment, it is essential for MVAPs to provide warmth and care during their interactions. In terms of emotional support needs, both older patients and their family members hoped that the MVAPs could accompany the patients to alleviate their anxiety by chatting with them and providing psychological comfort while waiting for treatment. This finding is in line with the report (33) that simple conversational interventions, including active listening and empathetic communication, significantly reduce anxiety among older patients during medical visits and improve overall visit satisfaction. However, it should be noted that the psychological comfort provided by MVAPs is fundamentally different from professional psychological counseling offered by licensed therapists. Research indicates that psychological counseling conducted by non-professionals may carry potential risks (34). This suggests that the psychological comfort provided by MVAPs should follow closely defined professional boundaries, avoiding interventions beyond their competence, particularly diagnostic questioning and the use of clinical terminology.

### 4.3 Growth needs: educational and developmental aspects

#### 4.3.1 Learning needs: the opposite reviews

This study showed that a key subtheme of the growth needs of outpatient older patients with chronic disease and their family members was the willingness to learn. More than half of the older patients expressed their willingness to learn about knowledge of their diseases and how to go through the medical consultations. This is consistent with the findings of Swor et al. (35), who found that 58.6% of older patients were willing to learn healthcare-related knowledge and skills and believed they were capable of implementing these skills. The theory of re-socialization for older patients suggests that (36) they can adapt to society and maintain their physical and mental health through positive learning behaviours. Therefore, MVAPs could use simple language and visuals aids to help older patients understand and retain information (36). Furthermore, the learning content must be practical: the information provided should not only be accurate but also applicable to the patients’ daily lives. For example, offering patients practical advice on improving their diet and exercise habits can enhance their self-management abilities.

In contrast, this study also indicated that most family members believed that older patients with chronic diseases did not need to learn. This perception may stem from family members’ stereotypes about older adults (37). Internalized stereotypes on age can influence family members’ perceptions and behaviours towards older patients unconsciously. This leads them to believe that older patients fail to cope with changes in society and are more dependent on others (38). MVAPs should encourage family members to recognize and support the willingness of older patients to learn, thereby boosting patients’ self-confidence and sense of self-worth. However, MVAPs alone may not be sufficient to eliminate these stereotypes, which requires a broader societal effort. Promoting intergenerational communication between younger and older generations could be an effective way to reduce the stereotypes (39).

#### 4.3.2 Learning contents: difference between the two populations

Another subtheme of the growth needs among older patients and family members was learning contents. The interviewees in this study believed that the learning contents should include both learning knowledge about diseases and how to complete medical consultation. This is a new finding that older patients could put forward detailed learning needs according to their physical conditions, while the needs expressed by family members were less specific and lacked in-depth understanding. The possible reason for this phenomenon is that family members give advice more from the perspective of a bystander, without the deep knowledge that older patients have about their own conditions.

### 4.4 Service optimization needs: efficiency and personalization

This study revealed that family members had a more urgent need to shorten the waiting time for outpatient consultations. Although the procedures of outpatient care vary in different countries, long waiting time for patients is a common and prominent problem worldwide (40). Mwanswila et al. (41) believed that optimizing the scheduling of outpatient appointments plays a crucial role in shortening the waiting time of patients. This requires MVAPs to coordinate appointment schedules effectively, ensuring that the waiting time of older patients is minimized as much as possible. This study found that family members were concerned about the transparency of MVA services. They sought to be fully informed about the older patient’s care and be better involved in their medical visits. Rosland et al. (42) indicated the importance of family member involvement in managing older patients with chronic diseases, as it can improve both prognosis and quality of life. The transparency of MVA services can be better guaranteed by recording while the older patients and family members receive the MVA services and the provision of feedbacks of the MVAPs after service.

The results of the interviews also showed that both the older patients and their family members required individualized service, according to physical and psychological conditions, as well as language barriers. The needs for targeted and differentiated accompanying services in this study are in line with the findings from Yang et al. (43). To meet these individualized requirements, MVA service institutions should enhance their recruitment criteria by preferentially selecting candidates with relevant medical backgrounds, such as nursing or allied health professionals. This approach ensures that MVAPs possess the requisite professional knowledge and competencies to meet the diverse needs of older patients. Furthermore, existing patented technologies for intelligent matching of MVAPs can be implemented to optimize service delivery. Specifically, aligning patients with the most suitable MVA provider based on medical specialization, language proficiency (44), and experiences can significantly improve service precision and effectiveness.

### 4.5 Family-centered care in MVA services

The results of this study coincided with the concept of family-centered care (FCC) emphasized by Hsu (45), as reflected in the desire of family members to be actively involved in older patients’ security, the transmission of medical advice, emotional support, encouragement of diseases education, and assistance with medication management and other aspects of disease management. The concept of family-centered care enables family members to effectively participate in the health management of older patients with chronic diseases, which greatly improves the health outcomes (46). Therefore, by encouraging family members to actively participate in the MVA services, the platform can empower them to contribute meaningfully to both the MVA service and disease management of older patients, thus promoting a better medical experience and higher quality of life for patients.

### 4.6 Aligning MVA services with global healthy ageing initiatives

Globally, the United Nations General Assembly has declared 2021–2030 the United Nations Decade of Action on Healthy Ageing, which brings together governments, international agencies, academic institutions, and professionals to foster coordinated and catalytic cooperation aimed at improving the lives of older adults, their families, and communities (47). The issue of difficult access to medical care for older patients are prominent, exposing the inadequacy of the global health system to respond to the specific needs of older population. This study focused on the MVA services, aiming to improve the experience and satisfaction of the older patients during medical treatment by refining service delivery and providing practical guidance for the healthcare system improvements.

### 4.7 Limitations

The results of the study are important for helping construct and improve the MVA service system and improving the medical experience of older patients. However, several limitations should be considered:

(a) All interviewees were from a single hospital, and most were local residents of Hangzhou, a city with above-average economic development in China. The geographical characteristic of the participants may result in their elevated expectations for MVA service quality, potentially limiting the generalizability of findings to economically underdeveloped regions.
(b) This study only included participants who were able to understand and clearly respond to the interview questions. As a result, the needs of participants with cognitive, audio-visual, or speech impairments were not addressed, which may limit the applicability of the findings to these vulnerable populations.
(c) As the study was conducted within Chinese socio-cultural context, the identified MVA needs may not fully align with requirements in other countries with differing healthcare systems and cultural norms.

In the future, qualitative research can be carried out in different countries, regions with different economic development levels. Additionally, the needs for MVA services of people with cognitive, audio-visual and speech disorders and their family members could be further explored. Thus, evidence support and action direction to meet the needs of the older population could be provided.

## 5 Conclusion

This study explored the needs of outpatient older patients with chronic diseases and their family members for MVA services. The findings revealed that their needs were diverse, covering not only basic medical assistance and companionship, but also emotional support, learning knowledge, and personalized MVA service needs. This result provides a reference for the construction of a more accurate MVA system for older patients with chronic diseases. The specific needs of patients with chronic diseases should be fully considered, which include existence needs, relatedness needs, growth needs, and needs for optimizing service provision. Plus, hospitals and MVA services institutions should improve the regulations of personnel management, conduct qualification assessment, to improve the quality of life for older patients and promote healthy ageing globally.

## Data Availability

The original contributions presented in the study are included in the article/Supplementary material, further inquiries can be directed to the corresponding author.
