# Supplementary material for: The needs for medical visit accompaniment services among older patients with chronic diseases and their family members: a qualitative study
Source: Front Public Health. 2025 May 9;13:1577329. doi: 10.3389/fpubh.2025.1577329 (PMC12098082; doi:10.3389/fpubh.2025.1577329)
Supplement: Supplementary file 1 [file Table_1.DOCX]

Supplementary Material

# 1.Supplementary Figures and Tables

## Supplementary Table

**Table 1 Consolidated criteria for reporting qualitative studies (COREQ): 32-item checklist**

| **Item** | **Guide questions/description** | **Answer** | **Page number** |
| --- | --- | --- | --- |
| Domain 1: Research team and reﬂexivity |  |  |  |
| Personal Characteristics |  |  |  |
| 1. Interviewer*/*facilitator | Which author*/*s conducted the interview or focus group? | CYH、ZJY、FQY、WCH | Page 5 |
| 2. Credentials | What were the researcher’s credentials? E.g. PhD, MD | Five were bachelor students, and one was a PhD | Page 5 |
| 3. Occupation | What was their occupation at the time of the study? | Five were bachelor students, and one was a lecturer in nursing school | Page 5 |
| 4. Gender | Was the researcher male or female? | Five females and 1 male | Page 5 |
| 5. Experiencing and training | What experience or training did the researcher have? | One researcher has rich experience in qualitative research; others are nursing students. They all have a history of training about how to conduct a qualitative study | Page 5 |
| Relationship with participants |  |  |  |
| 6. Relationship established | Was a relationship established prior to study commencement? | Yes | Page 4 |
| 7. Participant knowledge of the interviewer | What did the participants know about the researcher? e.g. personal goals, reasons for doing the research | The research aims, reasons for doing the research and what we want to improve among these people | Page 4 |
| 8.Interviewer characteristics | What characteristics were reported about the interviewer/facilitator? e.g. Bias, assumptions, reasons and interests in the research topic | We avoided assumptions before we collected and analyzed the interviewing data | Page 6 |
| Doman 2: study design |  |  |  |
| Theoretical framework |  |  |  |
| 9. Methodological orientation and Theory | What methodological orientation was stated to underpin the study? e.g. grounded theory, discourse analysis, ethnography, phenomenology, content analysis | Content analysis | Page 5 |
| Participant selection |  |  |  |
| 10. Sampling | How were participants selected? e.g. purposive, convenience, consecutive, snowball | Purposive | Page 3 |
| 11. Method of approach | How were participants approached? e.g. face-to-face, telephone, mail, email | Face-to-face | Page 4 |
| 12.Sample size | How many participants were in the study? | 19 older patients and 17 family members | Page 5 |
| 13. Non-participation | How many people refused to participate or dropped out? Reasons? | 5 people. Because it’s time for them to see the docotr or receiving therapy | Page 5-6 |
| Setting |  |  |  |
| 14. Setting of data collection | Where was the data collected? e.g. home, clinic, workplace | In the outpatient clinic | Page 4 |
| 15. Presence of non-participants | Was anyone else present besides the participants and researchers? | No | Page 4 |
| 16. Description of sample | What are the important characteristics of the sample? *e.g. demographic data, date* | Please see Table 1 and Table 2 | Page 6-11 |
| Data collection |  |  |  |
| 17.Interview guide | Were questions, prompts, guides provided by the authors? Was it pilot tested? | Yes. Yes | Page 5 |
| 18. Repeat interviews | Were repeat interviews carried out? If yes, how many? | No | Page 6 |
| 19. Audio/visual recording | Did the research use audio or visual recording to collect the data? | Yes. Audio recording | Page 5 |
| 20. Field notes | Were ﬁeld notes made during and/or after the interview or focus group? | Yes | Page 5 |
| 21. Duration | What was the duration of the interviews or focus group? | About 30 mins each | Page 5 |
| 22. Data saturation | Was data saturation discussed? | Yes | Page 4 |
| 23. Transcripts returned | Were transcripts returned to participants for comment and/or correction? | Yes | Page 6 |
| Domain 3: analysis and fingings |  |  |  |
| Data analysis |  |  |  |
| 24. Number of data coders | How many data coders coded the data? | 4. For the part of older patients, there were 2; for the part of family members, there were another 2 coders | Page 5 |
| 25. Description of the coding tree | Did authors provide a description of the coding tree? | Yes. Please see Figure 1 | Page 12 |
| 26. Derivation of themes | Were themes identiﬁed in advance or derived from the data? | Derived from the data | Page 6 |
| 27. Software | What software, if applicable, was used to manage the data? | MaxQDA 2022 | Page 5 |
| 28.Participant checking | Did participants provide feedback on the ﬁndings? | Yes | Page 5 |
| Reporting |  |  |  |
| 29. Quotations presented | Were participant quotations presented to illustrate the themes */* ﬁndings? Was each quotation identidied? E.g. participant number | Yes. Yes | Page 12-18 |
| 30. Data and findings consistent | Was there consistency between the data presented and the ﬁndings? | Yes | Page 12-18 |
| 31. Clarity of major themes | Were major themes clearly presented in the ﬁndings? | Yes | Page 12-18 |
| 32. Clarity of minor themes | Is there a description of diverse cases or discussion of minor themes? | Yes | Page 12-18 |

.
